# The Association Between Overweight Obesity Status and Hypertension in a Rural Community in Dang District, Gujarat, India: A Cross-Sectional Study

**DOI:** 10.7759/cureus.72030

**Published:** 2024-10-21

**Authors:** Christi M Navarro, Sneh Shah

**Affiliations:** 1 Public Health, Nova Southeastern University, Dr. Kiran C. Patel College of Osteopathic Medicine, Fort Lauderdale, USA; 2 Medicine, Nova Southeastern University, Dr. Kiran C. Patel College of Osteopathic Medicine, Fort Lauderdale, USA

**Keywords:** bmi, high blood pressure, hypertension, india, obesity, overweight and obesity, rural

## Abstract

Background

The relationship between overweight obesity status and hypertension is well-known throughout the world, especially in low socioeconomic communities and developing countries. Both high blood pressure and obesity are preventable risk factors for noncommunicable disease, death, and disability. The prevalence of obesity-overweight status in India is increasing faster than the global average, and diabetes in Southeast Asia has surged over the past few decades. There have been few systematic studies focusing on public health development in rural communities of India, like the Dang District. This study explores the association between overweight-obesity status and hypertension prevalence in a rural community in Dang district, Gujarat, India.

Methods

A cross-sectional design was utilized for this study, involving 1012 adult patient charts collected from medical camps in December 2018 and 2019. Patients with incomplete information for measurements of blood pressure, height, weight, age, and sex were omitted from the analysis (n=953). Data on BMI and blood pressure were analyzed to examine the relationship between overweight-obesity status and hypertension. Hypertension was defined by the American Heart Association (AHA) cut-offs for measured blood pressure. Both World Health Organization (WHO) and South Asian cut-offs were used for BMI. Binary logistic regression was used to assess the association between hypertension and overweight-obesity status, adjusted for age and sex.

Results

Most patients were hypertensive, with males having a higher prevalence (63.3%) than females (55.1%). The prevalence of hypertension among participants increased with age. This was true for both sexes, except for males 45-54 years of age. The average BMI for both sexes was 22.4. Results indicated that overweight and obese individuals had a significantly higher prevalence of hypertension compared to their normal-weight counterparts, suggesting a strong association between the two conditions. A binary logistic regression found that males were 1.35 times more likely to have hypertension than females (95% CI 1.03 - 1.79), and increasing age and BMI were associated with an increased likelihood of hypertension.

Conclusion

The association between hypertension and BMI is positive and is stronger when using South Asian cut-offs. Using these cut-offs will include a wider range of people at risk for hypertension. With males and older adults especially at risk, targeting public health awareness campaigns to reduce BMI and help lower the burden of hypertension can improve the health and quality of life in this community.

## Introduction

Hypertension, high blood pressure, and obesity are the leading indicators of poor health outcomes that impact quality of life and longevity [1]. These factors lead to many chronic non-communicable diseases related to poor cardiovascular and metabolic health [2-4]. Over the past few decades, chronic disease rates have increased and affect people across the world in low, middle, and high-income countries [5-7].

The social determinants of health help determine the quality of life of an individual. There are detailed components of the social determinant model that allow for public health and medical officials to examine key areas and plan both prevention and intervention programs [5]. The main components of social determinants of healthcare are education level, income, occupation, culture, and environment [8-9]. These are crucial underlying characteristics that identify an individual's socioeconomic status. Low socioeconomic status is associated with negative health outcomes and has higher mortality rates than those with higher socioeconomic status.

India is a low- and middle-income country (LMIC) that suffers due to the rising burden of chronic disease [10]. The rural Dang region of Gujarat faces significant challenges to overcome these public health issues. Over 75% of the Dang district population lives under the standard poverty line of Gujarat. The Kiran C. Patel College of Osteopathic Medicine (KPCOM) at Nova Southeastern University values commitment and diligent efforts to provide much-needed healthcare to underserved rural communities, both domestically and globally. Since 2014, KPCOM has deployed international medical outreach trips to a rural community in Ahwa in Dang, which provides health care services free of cost. These services aid in promoting the well-being and improve the quality of life of the citizens of Dang.

While previous studies have discussed the prevalence of overweight, obesity, and hypertension in the state of Gujarat [3, 11], there have been systematic and secondary analysis studies focusing on the public health development of rural Ahwa, especially those that focus on the association between obesity and hypertension in this area. Understanding the relationship between BMI and hypertension in this low socioeconomic area can provide insight into the burden of chronic disease, guiding effective medical and public health interventions. This study explores the association between overweight-obesity status and hypertension prevalence in a rural community in the Dang district of Gujarat, India.

This article was previously presented as a meeting abstract at the 2021 American Public Health Association Annual Meeting on October 22, 2021.

## Materials and methods

Study design and data source

This cross-sectional study analyzed medical records of adults presenting to medical outreach camps in the rural community of Ahwa in the Dang District in Gujarat, India. A retrospective chart review was completed for 1012 adult patients who presented to the medical camp in December 2018 and December 2019. Patients were included in the study if they were ≥18 years of age and medical charts had completed information for age, sex, and measurements of blood pressure, height, and weight (n= 953). A power analysis conducted determined that a sample size of 387 was sufficient to achieve a power of 0.80, given the medium effect size (O 1.8) and alpha level of 0.05. This study received approval from Nova Southeastern University's Institutional Review Board.

Measurement of body mass index (BMI)

Body mass index (BMI) was calculated by dividing weight in kg by height in meters squared (m^2^) [2,3]. The BMI for the World Health Organization (WHO) cut-off measurements for BMI were <18.5 kg/m^2^ as underweight, 18.5-24.9 kg/m^2^ as normal, 25.0-29.9 kg/m^2^ as overweight, and ≥30 kg/m^2^ as obese [12]. The recommended South Asian cut-off measurements were <18.0 kg/m^2^ as underweight, 18.0-22.9 kg/m^2^ as normal, 23.0-27 kg/m^2^ as overweight, and ≥27 kg/m^2^ as obese [13]. 

Blood pressure measurement and hypertension

Blood pressure was measured by trained medical students using standardized instruments and procedures. The cut-offs for hypertension were defined by the American Heart Association (AHA) [14]. Normal blood pressure was a systolic less than 120 mmHg and diastolic less than 80 mmHg. Elevated blood pressure had a systolic between 120-129 mmHg and a diastolic less than 80. High blood pressure (hypertension stage 1) had a systolic between 130-139 mmHg or diastolic between 80-89 mmHg. High blood pressure (hypertension stage 2) had a systolic higher than 140 mmHg or diastolic more than 90 mmHg. A hypertensive crisis was a systolic higher than 180 mmHg and/or diastolic higher than 120 mmHg [14]. For the purpose of this study, hypertension was defined as any individual that met the hypertension stage 1, stage 2, or hypertensive crisis criteria.

Statistical analysis

Descriptive statistics were stratified by sex using proportions for categorical variables (WHO and South Asian cut-offs for BMI; hypertension) and mean and standard deviation (SD) for continuous variables (age, calculated BMI). The age-specific prevalence of hypertension was also calculated for defined age groups. To examine the strength of the association between hypertension and overweight-obesity status using WHO and South Asian cut-offs for BMI, crude odds ratios were calculated to analyze the estimated strength of the association between BMI and hypertension. Binary logistic regression was used to assess the association between hypertension and overweight-obesity status for both WHO and South Asian BMI cut-offs, adjusted for age and sex. A p-value of <0.05 was set as statistically significant. All statistical analysis was performed using SPSS Statistics v.28 (IBM Inc., Armonk, New York).

## Results

A total of 953 men and women from the Awha region in the Dang District were included in this study, with completed blood pressure, height, and weight measurements. The study population was 56% female, and the mean age of the participants was 42.4 years.

Table 1 shows the distribution of BMI and blood pressure measurements for the study population. The average BMI for both sexes was 22.4. On average, females had higher BMI than males. According to both WHO (BMI >25) and South Asian (BMI >23) classification systems for overweight-obese, more females were overweight and obese (24.3% and 34.7%, WHO and South Asian, respectively) than males (21.0% and 29.8%, respectively). In contrast, males had a higher prevalence (63.3%) of hypertension (systolic >130 mmHg or diastolic >80 mmHg) than females (55.1%).

**Table 1:** Distribution of body mass index (BMI) levels and hypertension status among study population by sex Hypertension was defined as any individual that met the hypertension stage 1, stage 2, or hypertensive crisis criteria (i.e., systolic ≥130 mmHg or diastolic ≥80 mmHg).

Variables	Male	Female
Average BMI (SD)	22.4 (7.5)	22.4 (6.4)
BMI status (WHO), n (%)		
Underweight, <18.5	111 (26.7)	140 (26.1)
Normal, 18.5–24.9	218 (52.4)	272 (50.7)
Overweight, 25.0–29.9	61 (14.7)	76 (15.2)
Obese, ≥30.0	26 (6.3)	49 (9.1)
BMI status (Sout Asia), n (%)		
Underweight, <18.0	89 (21.4)	115 (21.4)
Normal, 18.0–22.9	203 (48.8)	236 (43.9)
Overweight, 23.0–27	79 (19.0)	103 (19.2)
Obese, ≥27	45 (10.8)	83 (15.5)
Hypertensive, n (%)		
Yes	264 (63.5)	296 (55.1)
No	152 (36.5)	241 (44.9)

**Figure 1:**
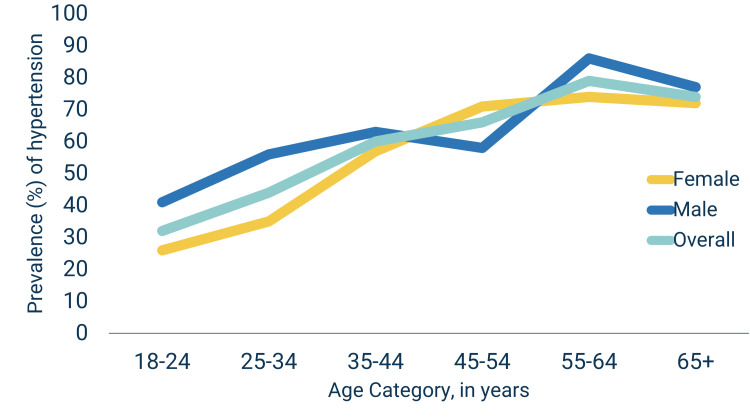
Age-specific prevalence of hypertension, overall and by sex

Figure 1 shows the distribution of age-specific prevalence of hypertension for males and females. Hypertension increased with age and ranged from 32.2% in the 18-24 year group to 79% in the 55-64 year group. Hypertension rates declined after age 65, and this trend was consistent for both sexes. For males, there was a decline in hypertension at age 45-54 years (58%) but it increased sharply at 55-64 years (86%) before declining again at age 65+ years (77%) (Figure 1).

**Figure 2:**
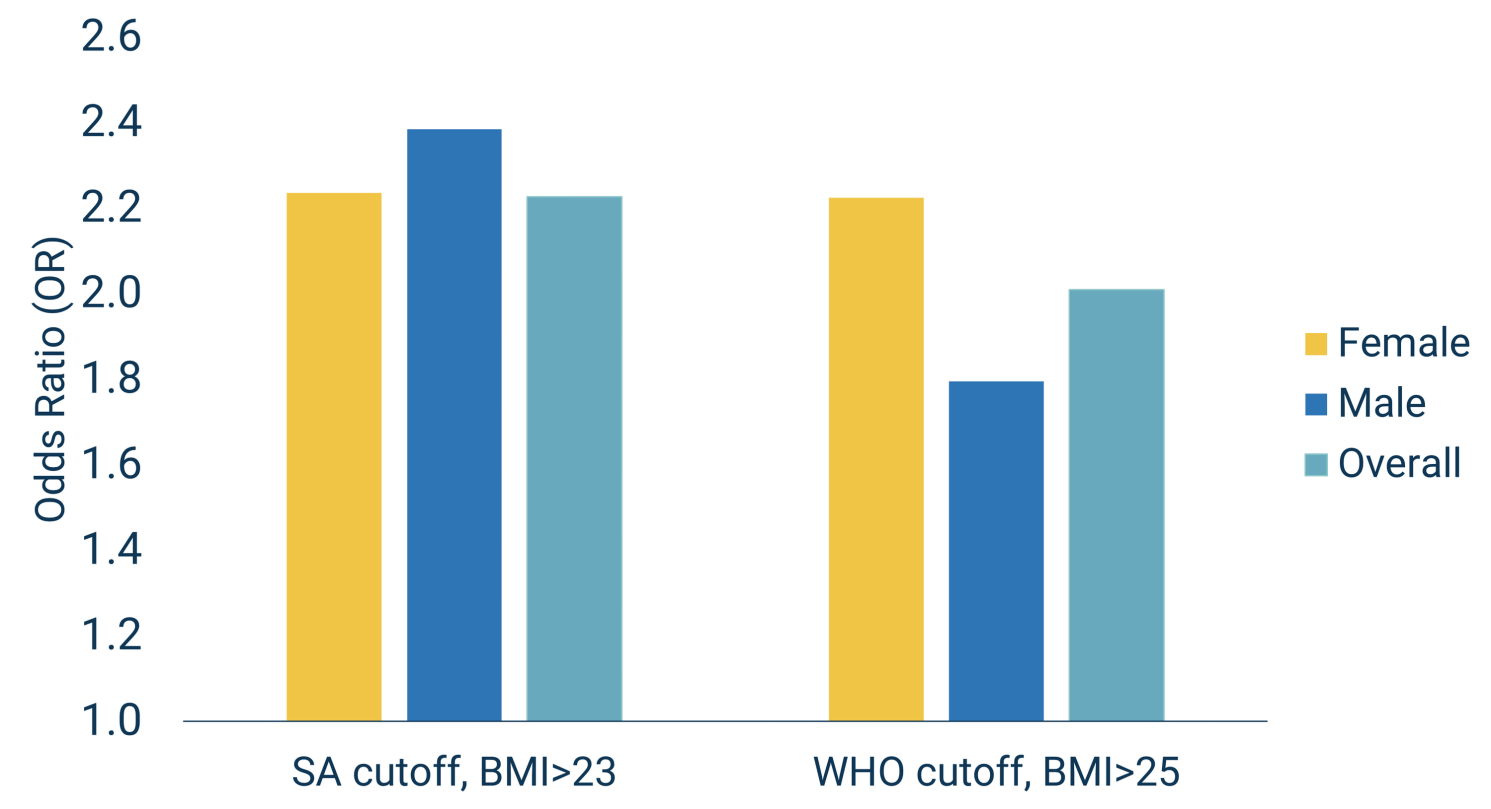
Odds ratios (ORs) of hypertension by overweight-obesity status, overall and by sex OR calculation = (#cases that are hypertensive/# cases that are not hypertensive) / (#controls that are hypertensive/# controls that are not hypertensive) case = BMI of ≥25 with WHO cut-offs; ≥23 with South Asian cut-offs control = BMI of <25 with WHO cut-offs; <23 with South Asian cut-offs

Odds ratios were computed to assess the association between hypertension and overweight-obesity status. Individuals with overweight and obese status had higher odds of having hypertension compared to individuals with normal or underweight status (Figure 2). Overweight-obese had over two-fold increase in the odds of hypertension. These findings were similar for both WHO (OR=2.01) and South Asian cut-offs (OR=2.26). Using WHO cut-offs, however, males showed a lower risk for hypertension (OR=1.8) than with South Asian cut-offs (OR=2.4). The risk for females was the same for both WHO and South Asian cut-offs (OR=2.2; Figure 2). When examining overweight and obesity status separately, overweight men were 2.3 times more likely to have hypertension than their normal-weight counterparts, whereas obese males were 2.1 times more likely.

Binary logistic regressions were performed to ascertain the effects of age, sex, and overweight-obesity status on the likelihood that participants have hypertension.

The logistic regression model using the South Asian cut-offs for overweight-obesity (BMI >23)was statistically significant, χ^2^(3) =126.37, p<0.001. The model explained 16.7% (Nagelkerke R^2^) of the variance in hypertension and correctly classified 66.4% of cases. Males were 1.35 times more likely to have hypertension than females. Increasing age was associated with an increased likelihood of hypertension, as compared to 18-24 year old individuals (reference). Individuals who were 55-64 years of age were 8.142 times more likely to have hypertension than their younger counterparts, and those who were over 64 years of age were 6.14 times more likely to have hypertension. Individuals with a BMI of 23 or greater were 2.18 more likely to have hypertension than those with a BMI <23 (Table 2).

**Table 2:** Crude odds ratios and adjusted odds ratios with 95% confidence intervals (CI) associated with hypertension, by age, sex, and overweight-obesity status AOR - adjusted odds ratio; COR - crude odds ratio; CI - 95% confidence interval

Variable	Hypertensive	COR (95% CI)	p-value	AOR (95% CI)	p-value
Age groups					
18-24 years	37 (32.2%)	-			
25-34 years	83 (43.7%)	1.635 (1.01 - 2.66)	0.047	1.545 (.95 - 2.53)	0.082
35-44 years	134 (59.6%)	3.104 (1.93 - 4.98)	< .001	2.76 (1.70 - 4.47)	< .001
45-54 years	127 (66.1%)	4.119 (2.52 - 6.74)	< .001	3.791 (2.30 - 3.25)	< .001
55-64 years	112 (79.4%)	8.142 (4.63 - 14.33)	< .001	7.506 (4.24 - 13029)	< .001
65+ years	637 (74.4%)	6.141 (3.32 - 11.35)	< .001	5.694 (3.06 - 10.59)	< .001
Sex					
Female	296 (52.9%)	1.41 (1.09 - 1.84)	0.01	1.35 (1.03 - 1.79)	0.032
Male	264 (47.1%)				
Overweight obese South Asian cut-offs (BMI >23)					
Yes	221 (39.5%)	2.26 (1.69 - 3.02)	< .001	2.157 (1.59 - 2.92)	< .001
No	339 (60.5%)				
Overweight obese WHO cut-offs (BMI > 25)					
Yes	151 (27.0%)	2.01 (1.44 - 2.80)	< .001	1.90 (1.35 - 2.68)	< .001
No	409 (73.0%)				

The logistic regression model using the WHO cut-offs for overweight obesity (BMI >25)was statistically significant, χ2(3) =114.84, p<0.001. Individuals with a BMI of 25 or greater were 1.90 more likely to have hypertension than those with a BMI <25.

## Discussion

Hypertension has a well-established association with obesity, significantly increasing the risk of cardiovascular and other chronic diseases. The World Health Organization (WHO) has deemed hypertension a global health crisis, reporting that 40% of adults aged 25 and older are diagnosed with hypertension [15]. In India, the prevalence of overweight-obesity status and hypertension is notably high but varies by state [3]. A recent cross-sectional study found an elevated prevalence of uncontrolled hypertension (60.2%) among adults in Gujarat [11]. This study, which focused on adults in the Dang District of Ahwa, a rural community in Gujarat, revealed an even higher hypertension prevalence (68.8%), particularly with increasing age and with variations by sex.

The positive associations between hypertension and overweight-obesity status, observed across different BMI cutoffs, were consistent in both sexes. Individuals with overweight-obesity status were more likely to have hypertension than those with normal or underweight BMI status. This aligns with previous studies on hypertension and overweight-obesity in India [2,3], where men aged 55-64 years exhibited higher hypertension rates, and individuals with elevated BMI had increased odds of developing hypertension. Notably, the overall hypertension prevalence in this study was higher than in previous research, suggesting unique or exacerbating factors in this rural population.

Several factors may explain the observed trends in hypertension and overweight obesity in this rural population. South Asians, regardless of economic status, have a stronger positive correlation between BMI and blood pressure than other ethnic groups [2-3]. The increasing prevalence of hypertension with age could be attributed to both biological aging and lifestyle factors, including physical inactivity, poor diet, and low health literacy. In Gujarat, the shift to more processed, calorie-dense foods due to food consumption and nutrition transitions may play a significant role in rising rates of obesity, diabetes, and malnutrition [16-17]. Additionally, reduced physical activity, possibly linked to shifts in occupational patterns (from agricultural work to more sedentary jobs), could contribute to weight gain and elevated blood pressure.

Previous studies on South Asian populations have observed wide variations in the prevalence of hypertension among subpopulations. For example, the Demographic and Health Survey across Bangladesh, Nepal, and India found hypertension rates at 10.1% [16]. The Annual Health Survey in India found that 19% of adult participants had hypertension [18]. The Longitudinal Ageing Study in India estimated hypertension prevalence at 41.9% among adults aged 45 years and older [19]. Additionally, Islam et al.'s machine learning model found that age and BMI were significant factors for hypertension.

The study's findings revealed sex-specific differences in the association between BMI and hypertension, consistent with other studies in South Asian populations. Hossain et al. [2] found similar trends in the sex-specific association between BMI and hypertension in South Asian populations. Using data from the Demographic and Health Survey (DHS) in Nepal, India, and Bangladesh, females had slightly higher BMI than males and a greater prevalence of overweight and obesity, consistent with our findings. A recent study also identified multiple sex-specific factors associated with the development of hypertension in women [20].

Biological sex impacts the underlying factors of chronic disease, such as fat tissue development [21-22]. For example, adipose (fat) tissue varies in amount depending on age, height, and weight, meaning that cardiovascular diseases (CVD) may be sex-sensitive. For instance, the risk for fatal coronary artery disease (CAD) associated with type 2 diabetes is much greater in women than in men with type 2 diabetes. A meta-analysis of 850,000 individuals at risk for CVD showed that women with diabetes had a 44% greater risk than men with diabetes [23].

This sex-specific difference is a critical gap when assessing the nature of non-communicable diseases (NCDs) in rural Ahwa. In the past decade, significant strides have been made in understanding the developmental differences in coronary artery disease (CAD) and ischemic heart disease (IHD) between sexes. High rates of BMI and hypertension may be the biggest indicators of CAD and IHD development in this population.

The strong association between obesity and hypertension observed in this study emphasizes the importance of routine BMI and blood pressure screening in rural healthcare settings. Clinically, the findings suggest that addressing obesity through lifestyle interventions, such as dietary modifications and increased physical activity, could reduce the risk of hypertension-related issues, including cardiovascular disease and stroke. From a public health perspective, these results highlight the urgent need for targeted obesity prevention and management programs in rural communities like Dang, a high-priority district for severe malnutrition [17]. Given the limited healthcare infrastructure in such areas, culturally tailored interventions focused on education, behavior change, and accessible healthcare services are critical to addressing this public health issue.

This study has several limitations. First, its cross-sectional design prevents the establishment of a causal relationship between BMI and hypertension. Second, incomplete or missing data in medical charts may have led to selection bias or errors in the recording and transcription of handwritten records. Third, the sample comprised individuals who sought primary care at the KPCOM medical camp, which may not be representative of the general population. Finally, the use of secondary data restricted the scope of the analysis, as we could not account for important variables such as diet, lifestyle, medications, or detailed medical history that could confound or mediate the observed associations.

Future research should consider longitudinal studies to establish causality between obesity and hypertension in rural populations. Additionally, expanding the study to include more comprehensive data, such as lifestyle factors, diet, and healthcare access, could offer deeper insights into the factors driving the high prevalence of hypertension and obesity in rural Gujarat. Finally, utilizing an electronic medical record method can increase the standardization, accuracy, and completion of patient notes.

## Conclusions

This study sought to examine the relationship between overweight obesity and hypertension in a rural community in Gujarat, India. The findings revealed a clear, positive association between BMI and hypertension rates. These results suggest that targeted interventions addressing overweight and obesity using South Asian BMI cut-offs are critical for reducing hypertension-related health risks in rural populations. Preventive measures and community health programs could significantly mitigate the growing public health burden. Future research could further investigate the long-term effects of obesity interventions on hypertension rates in similar underserved, rural communities.
